# DISRUPTION OF CPG ISLAND-MEDIATED CHROMATIN ARCHITECTURE AND TRANSCRIPTIONAL HOMEOSTASIS DURING AGING

**DOI:** 10.1093/geroni/igz038.756

**Published:** 2019-11-08

**Authors:** Samuel Beck, Junyeong Lee

**Affiliations:** MDI Biological Laboratory, Bar Harbor, Maine, United States

## Abstract

Aging causes the global disorganization of nuclear chromatin architecture. In a normal young nucleus, silent heterochromatin is associated with the nuclear lamina layer underlying nuclear envelope, thus spatially separated from euchromatin at the nuclear center. Notably, aging causes the disruption of nuclear lamina and the decondensation of associated heterochromatin. However, it is not clearly understood how these changes of chromatin architectures contribute to age-related diseases. Through large-scale computational analyses, we present that CpG islands (CGIs) give important clues to answering this question. CGIs are DNA elements with high Cytosine-phosphate-Guanine dinucleotide frequencies. In human, about 60% of total genes contain CGIs at their promoters (CGI+ genes) and are broadly expressed throughout the body. The other 40% of genes that do not have CGIs (CGI- genes) exhibit tissue-restricted expression patterns. Our results demonstrate that, in normal young nuclei, only CGI- genes can reside within lamina-associated heterochromatin when transcriptionally inactive, while CGI+ genes associate with nuclear central euchromatin even when they are repressed. In parallel, we show that age-associated heterochromatin decondensation can specifically de-repress tissue-specific CGI- genes leading to their uncontrolled expressions. Our results further demonstrate that global misregulation of CGI- genes increases the noise in gene transcription that, in turn, causes the loss of cellular identities during aging. Taken together, our study establishes critical implication of CGI-mediated chromatin architecture in age-associated degenerative changes and loss of tissue homeostasis.

